# Factors Associated With Predialysis Plasma Glucose Variability in Patients With Diabetes on Hemodialysis

**DOI:** 10.1155/ije/5465408

**Published:** 2026-07-30

**Authors:** Cheng-Chieh Yen, Chiung-Pei Huang, Hung-Pin Tu, Szu-Chia Chen

**Affiliations:** ^1^ Department of Internal Medicine, Division of Nephrology, Ditmanson Medical Foundation Chia-Yi Christian Hospital, Chiayi, Taiwan, cych.org.tw; ^2^ Department of Long-Term Care and Health Promotion, Min-Hwei College of Health Care Management, Tainan, Taiwan, mhchcm.edu.tw; ^3^ Hemodialysis Center, Ditmanson Medical Foundation Chia-Yi Christian Hospital, Chiayi, Taiwan, cych.org.tw; ^4^ Department of Public Health and Environmental Medicine, School of Medicine, College of Medicine, Kaohsiung Medical University, Kaohsiung, Taiwan, kmu.edu.tw; ^5^ Faculty of Medicine, College of Medicine, Kaohsiung Medical University, Kaohsiung, Taiwan, kmu.edu.tw; ^6^ Department of Internal Medicine, Division of Nephrology, Kaohsiung Medical University Hospital, Kaohsiung Medical University, Kaohsiung, Taiwan, kmu.edu.tw; ^7^ Department of Internal Medicine, Kaohsiung Municipal Siaogang Hospital, Kaohsiung Medical University, Kaohsiung, Taiwan, kmu.edu.tw

## Abstract

**Introduction:**

Patients with diabetes on hemodialysis (HD) have higher mortality and poorer health‐related quality of life than those without diabetes. In this setting, glycated hemoglobin is of limited value, whereas glycemic variability (GV) may better guide diabetes management and therapeutic monitoring. We aimed to identify factors associated with GV among our cohort.

**Methods:**

We retrospectively collected patient characteristics, dialysis parameters, and laboratory data of patients with diabetes on HD in 2024. GV was assessed by coefficient of variation (Glucose_cv) and variability independent of the mean (Glucose_VIM), derived from monthly predialysis random plasma glucose measurements. Associations between variables and GV were analyzed using regression and correlation methods.

**Results:**

After adjustment for confounders, age remained significantly and inversely associated with both Glucose_cv (*β* < −0.01, *p* = 0.02) and Glucose_VIM (*β* < −0.01, *p* = 0.02). In age‐stratified analyses, mean Glucose_cv differed significantly across quartiles (*p* < 0.01) and showed a significant decreasing trend (*p* < 0.01). Glucose_VIM likewise declined progressively across age quartiles with a confirmed downward trend (*p* < 0.01).

**Conclusions:**

Age showed an independent inverse relationship with GV in patients with diabetes on HD. These findings underscore the need for age‐tailored glycemic strategies in this patient population and support further studies to determine their impact on clinical outcomes.

## 1. Introduction

End‐stage renal disease (ESRD), defined by the need for renal replacement therapy (RRT) to sustain patient survival, has emerged as a significant global challenge for healthcare systems [1]. Hemodialysis (HD) remains the predominant RRT modality among ESRD patients, with diabetes mellitus (DM) accounting for approximately half of all ESRD cases globally [2]. Patients with diabetes on HD exhibit higher mortality rates and reduced health‐related quality of life compared to nondiabetes counterparts, primarily attributed to an increased burden of comorbid complications [3, 4]. This population imposes a considerable clinical and economic strain on healthcare systems [5].

In patients with diabetes and either normal renal function or nondialysis chronic kidney disease, improved glycemic control—as reflected by lower glycated hemoglobin (HbA_1c_) levels—has been consistently associated with a reduced incidence of diabetes‐related complications [6]. However, in patients undergoing HD, glucose homeostasis is significantly disrupted due to diminished renal gluconeogenesis, impaired insulin clearance, and the HD procedure itself, which facilitates the removal of both glucose and insulin. These factors contribute to pronounced glycemic fluctuations, including episodes of intradialytic hypoglycemia followed by postdialysis hyperglycemia [7–9]. Moreover, the utility of HbA_1c_ as a marker of glycemic control is limited in HD patients, owing to anemia, erythropoiesis‐stimulating agent administration, and shortened red blood cell lifespan [10, 11]. As a result, there is growing interest in the application of complementary metrics to better characterize glycemic patterns in this high‐risk cohort.

Glycemic variability (GV) describes fluctuations in glucose levels over time and includes both short‐term (intraday or day‐to‐day) and long‐term (visit‐to‐visit) variations. It is commonly assessed using metrics such as standard deviation, coefficient of variation (Glucose_cv), mean amplitude of glycemic excursions, and variability independent of the mean (Glucose_VIM), derived from plasma or capillary glucose measurements, as well as from data obtained through continuous glucose monitoring (CGM) or flash glucose monitoring systems [12]. Unlike HbA_1c_, which reflects average glycemia over a period of weeks to months, GV captures acute glucose fluctuations—including asymptomatic excursions—that may be overlooked by HbA_1c_ alone [13]. It is associated with elevated risks of complications and all‐cause mortality, highlighting its clinical significance not only in diabetes management [14] but also as a potential marker for monitoring therapeutic responses [15, 16]. Previous study has shown that Glucose_cv was positively associated with mortality risk in HD population [17], and Glucose_VIM had demonstrated a dose‐dependent association with the risk of stroke, myocardial infarction, and mortality in a large‐scale nationwide diabetic cohort study [18].

Understanding the clinical and dialysis‐related determinants of GV is crucial for effective risk stratification and individualized glycemic management in patients with diabetes on HD. However, evidence regarding determinants of GV in this population remains scarce. This study aims to identify factors associated with GV—quantified by the Glucose_cv and Glucose_VIM, derived from predialysis random plasma glucose measurements—in this cohort. Elucidating these associations may facilitate the development of targeted strategies to optimize glycemic control and mitigate related complications.

## 2. Material and Methods

### 2.1. Statement of Ethical Approval

This study was approved by the Institutional Review Board of Ditmanson Medical Foundation Chia‐Yi Christian Hospital (approval number: 2025106) on October 29, 2025. Given its retrospective design, the requirement for informed consent was waived. All procedures were performed in accordance with institutional guidelines and applicable ethical regulations [19].

### 2.2. Patients

Patient characteristics, dialysis parameters, and laboratory data were obtained from the Taiwan Society of Nephrology–Kidney Dialysis and Transplantation Registry. Collected patient characteristics included age, sex, HD vintage, comorbidities, and history of parathyroidectomy. Among the comorbidities, renal osteodystrophy was defined as the presence of abnormal mineral metabolism—such as disturbances in serum calcium, phosphate, vitamin D3, or parathyroid hormone levels—in conjunction with skeletal manifestations, including osteoporosis, chondrocalcinosis, or nontraumatic fractures [20]. The most recent HD parameters were recorded, including the arteriovenous access type, anticoagulant use, blood and dialysate flow rates, HD duration, dialyzer membrane surface area, and dialysate calcium concentration. Laboratory data obtained from predialysis blood samples were also collected for analysis. All patients in this study received HD using a dialysate solution containing 100 mg/dL of dextrose. The datasets generated and analyzed during the current study are available from the authors upon reasonable request.

### 2.3. Study Design

We retrospectively reviewed patients with diabetes undergoing maintenance HD at our hospital during the year of 2024. Patients with a dialysis vintage of less than 6 months (*n* = 18), those receiving dialysis via temporary catheters (*n* = 5), or those with incomplete data (*n* = 3) were excluded. Blood samples were obtained immediately before HD sessions although the interval between sampling and meal was not standardized. GV was assessed using two established metrics, both calculated from monthly predialysis random plasma glucose measurements recorded throughout 2024. The first was the Glucose_cv, defined as the standard deviation divided by the mean glucose level. The second was the Glucose_VIM, defined as the standard deviation divided by the mean glucose level raised to the power of *β*, where *β* is derived from the regression slope of the natural logarithm of the standard deviation on the natural logarithm of the mean.

### 2.4. Statistical Analysis

Statistical analyses were conducted using MedCalc Statistical Software (Version 23.3.7; MedCalc Software Ltd., Ostend, Belgium). Categorical variables were summarized as frequencies and percentages, whereas continuous variables were expressed as means with standard deviations. Associations between categorical variables and GV metrics such as Glucose_cv and Glucose_VIM were evaluated using general linear regression analysis. For continuous variables, Spearman’s rank correlation coefficients were calculated to assess their relationships with Glucose_cv and Glucose_VIM. Variables demonstrating significant associations in univariate analyses were subsequently included in multivariable regression models. Multiple comparisons among the quartiles were performed using one‐way analysis of variance followed by a Bonferroni post hoc test. Multicollinearity among independent variables was assessed using the variance inflation factor (VIF), with a VIF value of less than 10 considered indicative of no significant multicollinearity [21]. For significant continuous variables, subgroup analyses were performed by stratifying the cohort into quantiles. The Kruskal–Wallis test was used to assess statistical differences among the groups, while the Jonckheere–Terpstra trend test was applied to evaluate potential dose–response relationships across quartiles. A two‐tailed *p* value of < 0.05 was considered statistically significant.

## 3. Results

A total of 233 patients with diabetes receiving maintenance HD were included in the analysis for the year 2024. Patient characteristics are presented in Table 1. The mean age was 66 years, with males comprising 58% of the cohort. The most prevalent comorbidities included hypertension (98%), dyslipidemia (79%), coronary artery disease (61%), renal osteodystrophy (61%), congestive heart failure (57%), gout (55%), and gastrointestinal bleeding (53%). The average HD vintage was 7.1 years; 62% of the patients utilized arteriovenous fistulas, and 32% utilized arteriovenous grafts as their dialysis access. The mean predialysis random plasma glucose level was 179 mg/dL with a standard deviation of 45 mg/dL. The calculated GV metrics were 0.24 for Glucose_cv and 0.007 mg/dL for Glucose_VIM.

**TABLE 1:** Characteristics of the study population (*N* = 233).

Age (years)	66 ± 11	Albumin (g/dL)	4.0 ± 0.5
Sex (male)	58%	GPT (IU/L)	15 ± 11
Congestive heart failure	57%	Alkaline‐P (IU/L)	110 ± 62
Myocardial infarction	19%	Cholesterol (mg/dL)	149 ± 50
Cerebrovascular accident	25%	Triglyceride (mg/dL)	167 ± 146
Parathyroidectomy	17%	WBC (10^3^/μL)	6.5 ± 2.1
Hepatitis	26%	Hemoglobin (g/dL)	10.4 ± 1.1
Liver cirrhosis	17%	Hematocrit (%)	32.4 ± 3.3
Malignancy	20%	Platelet (10^3^/μL)	174 ± 63
Gastrointestinal bleeding	53%	Iron (μg/dL)	66 ± 32
Renal osteodystrophy	61%	TIBC (μg/dL)	225 ± 73
Asthma	1%	Ferritin (ng/mL)	598 ± 548
Cachexia	2%	TS (%)	30 ± 12
Hypertension	98%	BUN (mg/dL)	76 ± 22
Coronary artery disease	61%	Creatinine (mg/dL)	9.2 ± 2.3
COPD	7%	Sodium (meq/L)	137 ± 3
AIDS	< 1%	Potassium (meq/L)	4.6 ± 0.8
Gout	55%	Calcium (mg/dL)	9.2 ± 1.3
Dyslipidemia	79%	Phosphate (mg/dL)	4.9 ± 1.6
Dementia	7%	URR (%)	74 ± 0
HD vintage (years)	7.1 ± 5.6	Kt/V	1.6 ± 0.2
Type of AVA		PTH (pg/mL)	440 ± 397
AVF	62%	Glucose_mean (mg/dL)	179 ± 61
AVG	32%	Glucose_std (mg/dL)	45 ± 32
Anticoagulants	91%	Glucose_cv	0.24 ± 0.11
BFR (mL/min)	277 ± 34	Glucose_VIM (mg/dL)	0.007 ± 0.003
DFR (mL/min)	531 ± 92		
HD duration (hours)	4.0 ± 0.2		
Dialyzer MSA (m^2^)	1.8 ± 0.3		
DCC (mEq/L)	2.7 ± 0.4		

Abbreviations: AIDS, acquired immunodeficiency syndrome; Alkaline‐P, alkaline phosphate; AVA, arteriovenous access; AVF, arteriovenous fistula; AVG, arteriovenous graft; BFR, blood flow rate; BUN, blood urea nitrogen; COPD, chronic obstructive pulmonary disease; cv, coefficient of variation; DCC, dialysate calcium concentration; DFR, dialysate flow rate; GPT, glutamic pyruvic transaminase; HD, hemodialysis; MSA, membrane surface area; PTH, intact parathyroid hormone; std, standard deviation; TIBC, total iron binding capacity; TS, transferrin saturation; URR, urea reduction ratio; VIM, variability independent of the mean; WBC, white blood cell.

The regressions between categorical variables and GV metrics are presented in Table 2. Malignancy (*β* = −0.04, *p* = 0.03) was significantly negatively associated with Glucose_cv, whereas no categorical variable demonstrated a significant association with Glucose_VIM.

**TABLE 2:** Associations between categorical variables and glycemic variability metrics.

	Glucose_cv	*p* value	Glucose_VIM	*p* value
Sex (male vs. female)	−0.01	0.47	< −0.01	0.17
Congestive heart failure	< 0.01	0.90	< −0.01	0.44
Myocardial infarction	< 0.01	0.78	< 0.01	0.71
Cerebrovascular accident	< −0.01	0.84	< −0.01	0.85
Parathyroidectomy	−0.03	0.14	< 0.01	0.93
Hepatitis	< 0.01	0.77	< 0.01	0.45
Liver cirrhosis	0.02	0.36	< 0.01	0.24
Malignancy	−0.04	0.03	< −0.01	0.42
Gastrointestinal bleeding	−0.01	0.38	< −0.01	0.78
Renal osteodystrophy	< −0.01	0.93	< 0.01	0.15
Asthma	−0.04	0.61	< 0.01	0.96
Cachexia	−0.04	0.50	< −0.01	0.55
Hypertension	< 0.01	0.91	< 0.01	0.47
Coronary artery disease	< 0.01	0.75	< 0.01	0.81
COPD	0.03	0.29	< 0.01	0.11
AIDS	−0.01	0.92	< −0.01	0.57
Gout	0.01	0.49	< 0.01	0.44
Dyslipidemia	0.03	0.11	< 0.01	0.47
Dementia	< 0.01	0.85	< 0.01	0.87
AVA (AVF vs. AVG)	< −0.01	0.58	< 0.01	0.93
Anticoagulation	< −0.01	0.78	< −0.01	0.75

Abbreviations: AIDS, acquired immunodeficiency syndrome; AVA, arteriovenous access; AVF, arteriovenous fistula; AVG, arteriovenous graft; COPD, chronic obstructive pulmonary disease; cv, coefficient of variation; VIM, variability independent of the mean.

The correlations between continuous variables and GV metrics are presented in Table 3. Glucose_cv was significantly negatively correlated with age (*ρ* = −0.22, *p* < 0.01) and Kt/V (*ρ* = −0.14, *p* = 0.03), while blood flow rate demonstrated a significant positive correlation (*ρ* = 0.14, *p* = 0.04). Similarly, Glucose_VIM was significantly negatively correlated with both age (*ρ* = −0.21, *p* < 0.01) and Kt/V (*ρ* = −0.13, *p* = 0.04).

**TABLE 3:** Associations between continuous variables and glycemic variability metrics.

Variable	Glucose_cv	*p* value	Glucose_VIM	*p* value
Age (per 1 year)	−0.22	< 0.01	−0.21	< 0.01
HD vintage (per 1 year)	−0.12	0.07	< −0.01	0.99
BFR (per 1 mL/min)	0.14	0.04	0.11	0.10
DFR (per 1 mL/min)	0.04	0.55	0.02	0.71
HD duration (per 1 h)	0.09	0.19	0.08	0.20
Dialyzer MSA (per 1 m^2^)	0.05	0.43	0.07	0.27
DCC (per 1 mEq/L)	0.04	0.59	0.01	0.88
Albumin (per 1 g/dL)	−0.01	0.93	0.04	0.60
GPT (per 1 IU/L)	−0.04	0.51	−0.05	0.49
Alkaline‐P (per 1 IU/L)	0.07	0.29	0.10	0.12
Cholesterol (per 1 mg/dL)	< −0.01	0.96	0.01	0.88
Triglyceride (per 1 mg/dL)	0.10	0.12	< −0.01	0.97
WBC (per 10^3^/μL)	0.12	0.08	0.04	0.55
Hemoglobin (per 1 g/dL)	0.02	0.76	0.06	0.38
Hematocrit (per 1%)	0.02	0.79	0.06	0.36
Platelet (per 10^3^/μL)	0.06	0.38	−0.03	0.62
Iron (per 1 μg/dL)	−0.03	0.64	−0.02	0.74
TIBC (per 1 μg/dL)	0.05	0.41	0.03	0.64
Ferritin (per 1 ng/mL)	< −0.01	0.95	< −0.01	0.90
TS (per 1%)	−0.10	0.14	−0.07	0.29
BUN (per 1 mg/dL)	−0.06	0.37	−0.08	0.25
Creatinine (per 1 mg/dL)	−0.03	0.64	0.02	0.81
Sodium (per 1 mEq/L)	−0.12	0.06	0.04	0.56
Potassium (per 1 mEq/L)	−0.09	0.16	−0.08	0.26
Calcium (per 1 mg/dL)	0.01	0.94	< −0.01	0.94
Phosphate (per 1 mg/dL)	0.04	0.51	0.04	0.60
URR (per 1%)	−0.13	0.05	−0.12	0.08
Kt/V (per 1)	−0.14	0.03	−0.13	0.04
PTH (per 1 pg/mL)	0.07	0.32	0.09	0.16

Abbreviations: Alkaline‐P, alkaline phosphate; BFR, blood flow rate; BUN, blood urea nitrogen; cv, coefficient of variation; DCC, dialysate calcium concentration; DFR, dialysate flow rate; GPT, glutamic pyruvic transaminase; HD, hemodialysis; MSA, membrane surface area; PTH, intact parathyroid hormone; TIBC, total iron binding capacity; TS, transferrin saturation; URR, urea reduction ratio; VIM, variability independent of the mean; WBC, white blood cell.

Multivariable linear regression analyses were performed to further investigate the associations between GV metrics and variables identified as significant in the univariate analyses. After adjusting for potential confounders, age remained significantly and negatively associated with both Glucose_cv (*β* < −0.01, *p* = 0.02; Table 4) and Glucose_VIM (*β* < −0.01, *p* = 0.02; Table 5), indicating that advancing age is significantly correlated with reduced GV among patients with diabetes receiving maintenance HD. All included variables demonstrated no significant multicollinearity in the regression models.

**TABLE 4:** Multivariable analysis of factors associated with Glucose_cv.

Variables	*β*	SE	95% CI	*p* value	VIF
Constant	0.42	0.11	0.21–0.63	< 0.01	
Malignancy	−0.03	0.02	−0.07–0.01	0.14	1.05
Age (per 1 year)	< −0.01	< 0.01	< −0.01‐< −0.01	0.02	1.19
BFR (per 1 mL/min)	< 0.01	< 0.01	< −0.01‐< 0.01	0.87	1.16
Kt/V (per 1)	−0.05	0.03	−0.11–0.02	0.16	1.11

Abbreviations: BFR, blood flow rate; CI, confidence interval; SE, standard error; VIF, variance inflation factor.

**TABLE 5:** Multivariable analysis of factors associated with Glucose_VIM.

Variables	*β*	SE	95% CI	*p* value	VIF
Constant	0.01	< 0.01	0.01–0.02	< 0.01	
Age (per 1 year)	< −0.01	< 0.01	< −0.01‐< −0.01	0.02	1.07
Kt/V (per 1)	< −0.01	< 0.01	< −0.01‐< 0.01	0.15	1.07

Abbreviations: CI, confidence interval; SE, standard error; VIF, variance inflation factor.

**FIGURE 1:**
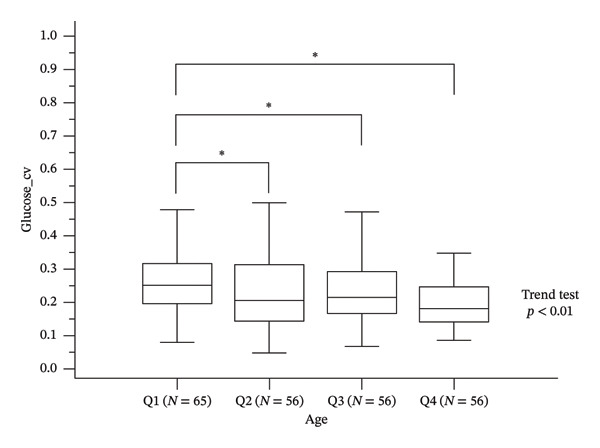
Subgroup analysis of age quartiles and Glucose_cv. Age quartiles were defined as follows: Quartile 1 (25–60 years), Quartile 2 (61–66 years), Quartile 3 (67–74 years), and Quartile 4 (75–91 years). cv: coefficient of variation; ^∗^ *p* < 0.05.

**FIGURE 2:**
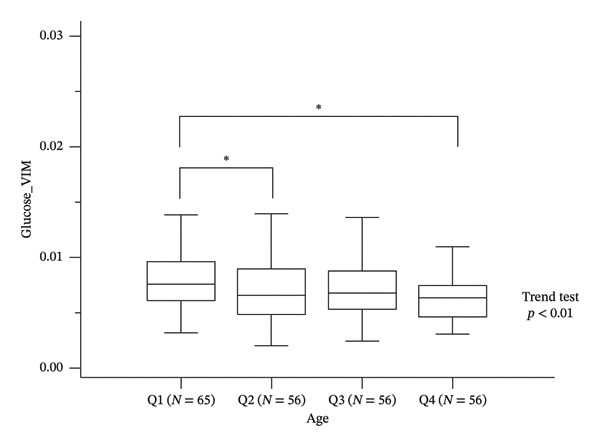
Subgroup analysis of age quartiles and Glucose_VIM. Age quartiles were defined as follows: Quartile 1 (25–60 years), Quartile 2 (61–66 years), Quartile 3 (67–74 years), and Quartile 4 (75–91 years). VIM: variability independent of the mean; ^∗^ *p* < 0.05.

Subgroup analyses of age were conducted by stratifying patients into quartiles. The mean Glucose_cv values across quartiles 1–4 were 0.27 ± 0.12, 0.23 ± 0.14, 0.23 ± 0.10, and 0.20 ± 0.08, respectively, demonstrating a statistically significant difference among groups (*p* < 0.01). A trend test revealed a significant decreasing pattern across quartiles (*p* < 0.01; Figure 1). Similarly, Glucose_VIM values decreased progressively across age quartiles, with means of 0.008 ± 0.003 mg/dL, 0.007 ± 0.004 mg/dL, 0.007 ± 0.003 mg/dL, and 0.006 ± 0.003 mg/dL, respectively (*p* < 0.01). The trend test also confirmed a significant downward trend (*p* < 0.01; Figure 2).

## 4. Discussion

In this study, GV, calculated using the Glucose_cv and Glucose_VIM derived from predialysis random plasma glucose measurements, demonstrated a significant inverse association with age in patients with diabetes on HD. These findings emphasize the necessity of age‐tailored strategies for glucose monitoring and management within this high‐risk population.

The Glucose_cv among study participants was 0.24 ± 0.11, based on random plasma glucose measurements collected prior to HD sessions over a 1‐year period. In comparison, Yusof Khan et al. [22] reported Glucose_cv values of 0.306 on HD days and 0.229 on non‐HD days in a cohort of 93 DHD patients, using 11‐point and 7‐point self‐monitoring blood glucose profiles, respectively. Furthermore, most published studies have calculated Glucose_cv using 24‐h CGM, with reported values ranging from 0.16 to 0.45 [11, 17, 23–26]. Despite differences in sampling frequency and methodology, our findings are broadly consistent with those reported in the literature. The Glucose_VIM in our cohort was 0.007 ± 0.003 mg/dL. In contrast, Echouffo‐Tcheugui et al. [27] conducted a post hoc analysis from the antihypertensive and lipid‐lowering treatment to prevent heart attack trial, in which Glucose_VIM based on fasting plasma glucose measured at three time points ranged from 0 to 258.7 mg/dL. Similarly, Kaze et al. [28] evaluated long‐term GV in HbA_1c_ and fasting plasma glucose across four visits in patients with Type 2 DM, reporting Glucose_VIM values ranging from < 0.05 to > 0.12 mg/dL. The marked differences in Glucose_VIM between our study and prior reports may be attributed to heterogeneity in study populations, variability definitions, and glucose measurement timings.

To further explore the determinants of GV, we conducted multivariable regression analyses incorporating variables that showed significance in the univariate models. After controlling for potential confounding factors, age persisted as an independent predictor and exhibited a significant inverse relationship with both Glucose_cv and Glucose_VIM. Our findings are in line with those reported by Rhee et al. [29], who analyzed data from 16,297 adults with DM initiating HD between 2006 and 2011 using the U.S. Renal Data System and U.S. Census data. In that study, younger age was independently associated with poor glycemic control. In contrast, Shi et al. [17] examined the relationship between GV—assessed via 48‐h CGM—and all‐cause mortality in 1240 patients with diabetes on HD and reported no significant difference in age across quartiles of Glucose_cv. In the aforementioned study by Yusof Khan et al. [22], Glucose_cv was derived from random blood glucose profiles. Their multivariable analysis demonstrated that age was positively significantly associated with above‐target Glucose_cv on HD days. These divergent observations across studies may be attributed to differences in the timing and modality of glucose sampling.

We propose several mechanisms to explain the observed inverse association between age and GV. First, age‐related declines in glycogen metabolism and hormonal responses—including insulin and counter‐regulatory hormones—may contribute to lower GV in older adults. Supporting this hypothesis, Curl et al. [30] investigated glucose kinetics and metabolic flexibility in healthy young and older people using oral glucose tolerance tests. Their findings demonstrated that glucose kinetics were significantly blunted in older individuals compared to their younger counterparts, indicating an age‐associated reduction in dynamic glucose handling. In addition, Jin et al. [31] analyzed CGM data alongside surrogate markers of insulin sensitivity and residual *β-*cell function—including fasting C‐peptide levels and body mass index (BMI)—in a cohort of 81 patients with Type 1 diabetes and 399 patients with Type 2 diabetes. Their findings indicated that greater GV was associated with higher insulin sensitivity and preserved insulin secretion. Second, younger patients with diabetes may be more likely to exhibit irregular mealtime patterns, contributing to greater GV. Shimizu et al. [32] conducted a cross‐sectional study involving 4421 individuals with diabetes aged 20–74 years, assessing self‐reported mealtime behaviors. Their findings revealed that irregular mealtimes were significantly associated with poorer glycemic control in women with Type 1 diabetes and with higher rates of obesity in men with Type 2 diabetes. Moreover, late dinner timing was associated with elevated HbA_1c_ levels and increased BMI among individuals with Type 2 DM. Third, older patients may exhibit higher levels of treatment adherence, which could contribute to reduced GV. In a descriptive cross‐sectional study of 133 elderly patients with Type 2 DM, Nascimento et al. [33] reported a high medication adherence rate of 97.7%. Similarly, in a large retrospective cohort study involving over 2,00,000 individuals treated with noninsulin antidiabetic medications, Kirkman et al. [34] observed that younger patients, those newly diagnosed with diabetes, and those prescribed fewer concurrent medications were more likely to exhibit poor adherence. Lastly, among older patients, those who survive and remain on maintenance HD represent a survivor cohort characterized by more favorable glycemic control and reduced GV. These individuals may inherently possess traits such as better treatment responsiveness or fewer comorbidities, which contribute to more stable glycemic profiles. Consequently, this selection bias may partially explain the inverse association observed between age and GV.

Studies focusing on GV in patients with diabetes on HD remain limited. Given the restricted accessibility of CGM due to cost constraints and the inaccuracy of HbA_1c_ as a glycemic marker in this population, our study utilized routine predialysis laboratory glucose measurements to assess GV and its associated factors. This approach offers a feasible and cost‐effective alternative for evaluating glycemic fluctuations in clinical practice including adjusting insulin dosage, selecting glucose‐lowering agents with lower hypoglycemia risk, modifying dietary counseling, or increasing the frequency of glucose monitoring in high‐risk patients. In addition, identifying age‐related differences in GV may assist clinicians in tailoring glycemic targets and therapeutic intensity according to patient characteristics. These findings may contribute to the development of individualized and applicable glucose management strategies in patients with diabetes on HD.

Several limitations of this study should be acknowledged. First, this was a single‐center analysis, which may limit the generalizability of our findings to broader patient populations. Second, data regarding diabetes duration and types of diabetes were unavailable in our cohort. These factors may have acted as potential confounders, as patients with Type 1 diabetes are generally younger and tend to exhibit higher GV [31]. Third, HbA_1c_ values, CGM data, and glucose measurements obtained on non‐HD days were unavailable in this study. Therefore, external validation of our GV metrics derived from predialysis plasma glucose measurements—which are less commonly utilized in clinical practice—could not be performed. Fourth, the current study did not explore the associations between GV and clinical outcomes such as complications, hospitalization, or mortality. Finally, the low *β* coefficients and *R* ^2^ values observed in our regression models suggest the presence of unmeasured confounding variables, including lifestyle factors, dietary patterns, and medication use, which were not captured in our dataset. Future studies involving larger, multicenter cohorts with more comprehensive data are warranted to validate these findings.

## 5. Conclusions

We observed that age was independently and inversely associated with GV derived from predialysis random plasma glucose measurements in patients with diabetes on HD. These results highlight the importance of age‐specific glycemic management in this high‐risk population. Further research is warranted to evaluate the effectiveness of such strategies in reducing GV and improving clinical outcomes.

## Funding

The authors received no financial support for the research, authorship, and publication of this article.

## Disclosure

A portion of this work was previously presented in a poster form at the 23^th^ Asian Pacific Congress of Nephrology (APCN 2025) [35].

## Conflicts of Interest

The authors declare no conflicts of interest.

## Data Availability

The data used to support the findings of this study are available on request from the corresponding author.
